# Expression of the Shrimp *wap* gene in *Drosophila* elicits defense responses and protease inhibitory activity

**DOI:** 10.1038/s41598-018-26466-6

**Published:** 2018-06-08

**Authors:** Dianxiang Li, Yuanyuan Luan, Lei Wang, Mei Qi, Jinxing Wang, Jidong Xu, Badrul Arefin, Meixia Li

**Affiliations:** 1 Biotechnology Department, School of Biological Sciences and Biotechnology, University of Jinan, Jinan, 250022 China; 2 Shandong Provincial Key Laboratory of Animal Cells and Developmental Biology, School of Life Sciences, Shandong University, Jinan, 250100 China; 3 Department of Molecular Biosciences, The Wenner-Gren Institute (MBW), Stockholm University, 10691 Stockholm, Sweden; 4 State Key Laboratory of Brain and Cognitive Science, Institute of Biophysics, Chinese Academy of Sciences, Beijing, 100101 China

## Abstract

The *wap* gene encodes a single whey acidic protein (WAP) domain-containing peptide from Chinese white shrimp (*Fenneropenaeus chinensis*), which shows broad-spectrum antimicrobial activities and proteinase inhibitory activities *in vitro*. To explore the medical applications of the WAP peptide, a *wap* gene transgenic *Drosophila melanogaster* was constructed. In *wap*-expressing flies, high expression levels of *wap* gene (>100 times) were achieved, in contrast to those of control flies, by qRT-PCR analysis. The *wap* gene expression was associated with increased resistance to microbial infection and decreased bacterial numbers in the flies. In addition, the WAP protein extract from *wap*-expressing flies, compared with control protein extract from control flies, showed improved antimicrobial activities against broad Gram-positive and Gram-negative bacteria, including the clinical drug resistant bacterium of methicillin-resistant *S*. *aureus* (*MRSA*), improved protease inhibitor activities against crude proteinases and commercial proteinases, including elastase, subtilis proteinase A, and proteinase K *in vitro*, and improved growth rate and microbial resistance, as well as wound-healing in loach and mouse models. These results suggest that *wap*-expressing flies could be used as a food additive in aquaculture to prevent infections and a potential antibacterial for fighting drug-resistant bacteria.

## Introduction

The increasing frequency of bacteria that are resistant to conventional antibiotics has led to increased infection and mortality rates and become a tremendous global public-health problem^1–3^. Therefore, discovering new-generation antimicrobials is urgent^4,5^.

Antimicrobial peptides (AMPs) are small peptides produced by various organisms as part of their innate immune responses^6–9^. Most AMPs are positively charged and hydrophobic peptides, which allow them to adopt an amphipathic structure and interact with the membrane of the microbes and destroy it^4,10^. Therefore, developing resistance against AMPs is not easy for microbial targets^11^. Currently, more than 3000 naturally occurring and synthetic AMPs have been reported^12,13^, and many of them have potential for the development of antimicrobial drugs, such as *Cecropin*, *Defensin*, and *Apidaecin*^14–17^. In addition to antimicrobial abilities, some of them display antitumor activity^18^. Based on the antimicrobial activities against a wide range of pathogens and the ability to defeat multidrug-resistant bacteria, AMPs are becoming popular as effective alternatives to current antibiotics^4,19^. However, successfully applying many AMPs in biomedical fields is very difficult due to their enzymatic degradation, expensive production and antigenicity *in vivo*^6,20^.

It just so happens that the *F*. *chinensis* WAP recombinant protein showed broad-spectrum antimicrobial and proteinase inhibitory activities^21^, which raised interest in developing WAP into a novel therapeutic agent against pathogens in clinical applications.

The GAL4/UAS system is routinely used to analyze the function of transgenes in *D*. *melanogaster*, and allows directed expression of an upstream activation sequence (UAS)-linked transgene by binding the yeast transcriptional activator (GAL4) that is placed under the control of a specific selectable promote^22^. In this study, several UAS-*wap* transgenic *D*. *melanogaster* strains were constructed by *w*^1118^ embryo microinjection technology. Meanwhile, the expression levels and antibacterial functions of *F*. *chinensis wap* gene in transgenic flies were studied. Additionally, the application of *wap*-expressing flies in aquaculture and anti-infection drug development was also analyzed.

## Results

### Construction of UAS-*wap* transgenic *D*. *melanogaster*

According to the constructing methods (see page 8 in Materials and Methods), an open reading frame (ORF) fragment of *F*. *chinensis wap* gene was inserted to pUAST vector to generate the *wap*/pUAST recombinant plasmid confirmed by enzyme digestion and sequencing (Supplementary Fig. S1). The *wap*/pUAST together with PΔ2–3 plasmid was co-injected into *D*. *melanogaster* embryos of *w*^1118^ to generate 10 red-eyed UAS-*wap* transgenic *D*. *melanogaster* lines, and the transformation rate was about 2.0% (Table 1). The UAS-*wap* transgenic strains were confirmed carrying *wap* gene by PCR using primers of *wap* F and *wap* R (Supplementary Fig. S2). Primers used in this study were listed in Table 2.

**Table 1:** Screening result of UAS-*wap* transgenic flies.

**injected embryos**	**hatched larvae**	**eclosion** **flies**	**transgenic flies**	hatched rate	eclosion rate	transformation rate
486	309	249	10	63.6%	80.6%	2.0%

**Table 2:** Primers used in this study.

**Primer**	**Sequence** **(5′-3′)**
*wap* F	GGGGTACCATGGTGAACATCAAGGAAGTTC
*wap* R	GCTCTTCTAGATTTTCCGTAGGGAGATCCCA
*wap-EX* F	CCGGAATTCATGGTGAACATCAAGGAAGTTC
*wap-EX* R	CCGCTCGAGTCATTTTCCGTAGGGAGATC
Smart F	TACGGCTGCGAGAAGACGACAGAAGGG
Oligoanchor R	GACCACGCGTATCGATGTCGACT_16_(A/C/G)
*attA* F	CCGAGGCACTTCCTTCACTT
*attA* R	GACCGCTTTGAGTGTTTCCG
*attB* F	CACAACTGGCGGAACTTTGG
*attB* R	CATTGCGCTGGAACTCGAAG
*dpt* F	ATTGCCGTCGCCTTACTTTG
*dpt* R	AATCTCGTGGCGTCCATTGT
*def* F	GCTCAGCCAGTTTCCGATGTG
*def* R	TCGTTGCAGTAGCCGCCTTG
*dro* F	CCACCACTCCAAGCACAATG
*dro* R	CAGCTTGAGTCAGGTGATCCT
*ce-A*_1_ F	ACATCTTCGTTTTCGTCGCTC
*ce-A*_1_ R	GCAGTTGCGGCGACATT
*ce-A*_2_ F	ACATCTTCGTTTTCGTCGCTC
*ce-A*_2_ R	GTTAACCTCGAGCAGTGGCT
*rp49* F	GCTTCAAGATGACCATCCGCCC
*rp49* R	GGTGCGCTTGTTCGATCCGTAAC
*β-actin* F	CAGGCGGTGCTTTCTCTCTA
*β-actin* R	AGCTGTAACCGCGCTCAGTA

### Mapping of *wap* gene

According to the mapping procedure (Supplementary Fig. S3), the males (♂UAS-*wap*, red-eyed) were selected from transgenic lines designated as t6, tx5, t10, t32, and t78 and implemented in two rounds of successive crosses with white-eyed 3703 balancer virgin females (☿w−/w−;Sco/Cyo;Sb/Tb) by 1♂:1☿ ratio. Results showed that the *wap* gene was located on chromosome X in t32 and t78 transgenic lines, and chromosome II of t6 transgenic line, and double-inserted at chromosomes II and III of tx5 and t10 transgenic lines, respectively.

### Expression profiles of the *wap* gene in *D*. *melanogaster*

**Figure 1:**
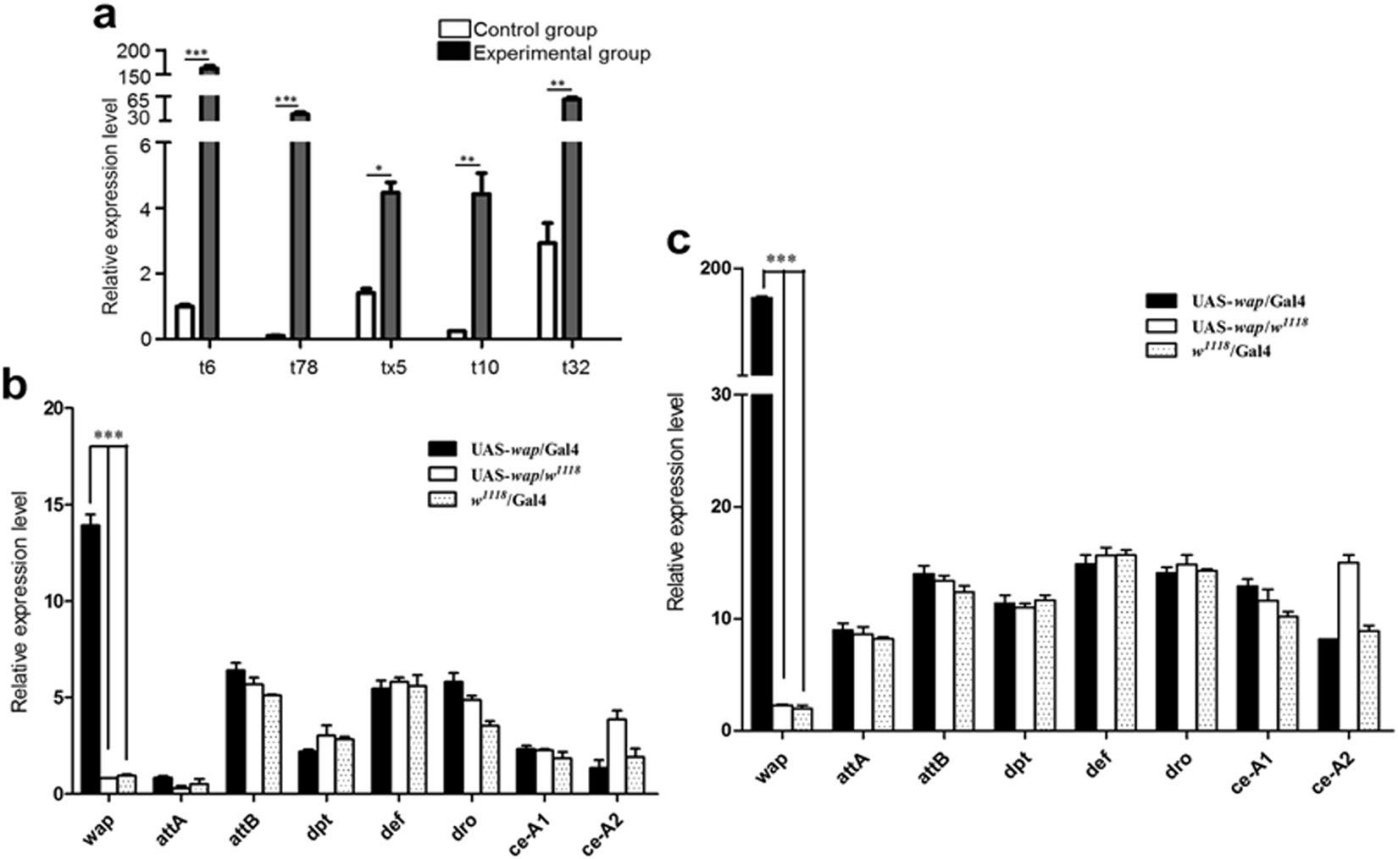
Gene expression profiles of *wap* and other AMPs in transgenic *D*. *melanogaster* detected by qRT-PCR. (**a**) The *wap* mRNA levels in the experimental and control groups were expressed as the ratio of *wap* to *β-actin*. The experimental group consisted of UAS-*wap/*Gal4 hybrids from crosses of t6, tx5, t10, t32, and t78 UAS-*wap* transgenic lines with *actin*-Gal4 line. The control group consisted of UAS-*wap*/*w*^1118^ hybrids from crosses of t6, tx5, t10, t32, and t78 with *w*^1118^, and *w*^1118^/Gal4 hybrids from crosses of *w*^1118^ with *actin*-Gal4. (**b**,**c**) mRNA levels of *wap* and known *D*. *melanogaster* AMP genes in experimental and control groups under normal conditions and *E*. *coli*-challenged for 24 h. *Rp49* was the internal control. The experimental and control groups were from the crosses of t6 with *actin*-Gal4, t6 with *w*^1118^ and *actin*-Gal4 with *w*^1118^, respectively. The error bars represent ± SD of three independent qRT-PCR amplifications and quantifications. The asterisks indicate significant differences (*p < 0.05; **p < 0.01; ***p < 0.001) between experimental and control samples.

For detecting the expression profiles, the UAS-*wap* transgenic strains of t6, tx5, t10, t32, and t78 were individually crossed with *actin*-Gal4 line. The UAS-*wap* transgene was driven by *actin*-Gal4 to express in all tissues of their progeny of UAS-*wap*/Gal4 (experimental group). The quantitative real-time polymerase chain reaction (qRT-PCR) showed that the expression levels of *wap* gene were very high in experimental groups, and maximal expression was observed in t6 experimental group by 160 times higher than that of control group of UAS-*wap*/*w*^1118^ from crosses of t6 UAS-*wap* transgenic line with *w*^1118^ line. The *wap* expression levels in t32 and t78 experimental groups were also high (close to 100 times). However, the *wap* expression level was undetectable in another control group of *w*^1118^/Gal4 from crosses of *actin*-Gal4 with *w*^1118^ (Fig. 1a). In addition, the expression levels of known *D*. *melanogaster* AMP genes, including *attacin A* (*attA*), *attacin B* (*attB*), *diptericin* (*dpt*), *defensin* (*def*), *drosocin* (*dro*), *cecropin A1* (*ce-A1*), and *cecropin A2* (*ce-A2*) were also examined together with *wap* gene in t6 experimental and control groups under normal culture conditions and bacterial infection by qRT-PCR. Results showed that the expression level of *wap* gene in experimental group was significantly higher than that of control groups, whereas the expression levels of known *D*. *melanogaster* AMP genes had no significant difference between experimental and control groups (Fig. 1b,c).

### Assessment of antibacterial activity on *wap*-expressing transgenic *D*. *melanogaster*

**Figure 2:**
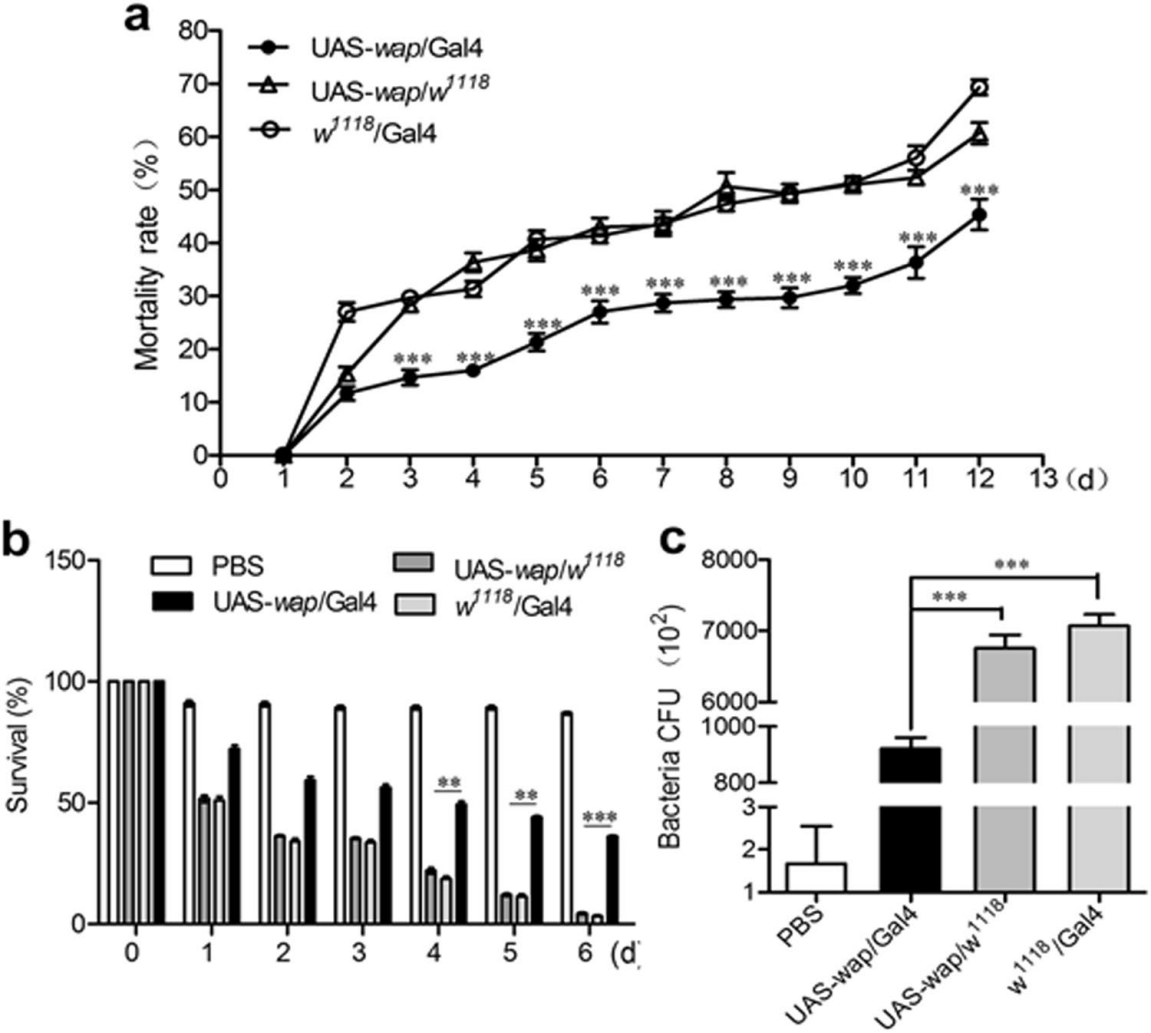
Assessment of antibacterial activity of *wap*-expressing transgenic *D*. *melanogaster*. (**a**) The mortalities of UAS-*wap/*Gal4, UAS-*wap/w*^1118^, and *w*^1118^*/*Gal4 flies fed with standard cornmeal/agar medium with *E*. *coli*, *S*. *aureus*, and *M*. *luteus* at 12 days after eclosion. (**b**) Survival rates of UAS-*wap/*Gal4, UAS-*wap/w*^1118^, and *w*^1118^*/*Gal4 flies for 6 days of post-*E*. *coli* infection. (**c**) Bacterial cfus in survived UAS-*wap/*Gal4, UAS-*wap/w*^1118^, and *w*^1118^*/*Gal4 flies after 6 days of post-*E*. *coli* infection. The error bars represent ±SD of three repeated experiments, and the asterisks indicate significant differences.

By feeding standard cornmeal/agar medium in the presence of *Escherichia coli*, *Staphylococcus aureus*, and *Micrococcus luteus* to parent crosses until their F1 progeny, experimental and control groups, reached adulthood, the antibacterial activities of *wap*-expressing flies were evaluated. Results showed that the mortalities of control groups were 2-folds higher than that of experimental group during 3–12 days of post-eclosion, but no significant difference was detected in the control groups (Fig. 2a). Furthermore, the survival rate of experimental group challenged by *E*. *coli* was significantly high compared with those of control groups at 4–5 days (*p* < 0.01), and 6 days (10 folds, *p* < 0.001) of post-injection. Notably, no significant difference was detected during 1–6 days of post phosphate buffered saline (PBS)-challenged experimental group (Fig. 2b). The results from *S*. *aureus* or *M*. *luteus–*challenge were similar to those in Fig. 2b (data not shown). Additionally, bacterial colonies were counted using surviving flies after 6 days of post-*E*. *coli* challenge. Results showed that the bacterial counts in experimental group were significantly decreased compared with those in control groups (*p* < 0.001) (Fig. 2c). The expression of *wap* gene endowed experimental flies with antibacterial activity against *E*. *coli*, which reached approximately 86% bacteriostasis rate.

### Monitoring the antimicrobial activity of WAP protein extract

WAP protein extract was prepared using UAS-*wap*/Gal4 experimental flies, and the control protein extract was prepared using control flies of UAS-*wap/w*^1118^ and *w*^1118^*/*Gal4. The total protein content was about 30 μg/mL in these protein extracts (1 mg/mL). Western blot analysis showed that, compared to control protein extract, the WAP protein extract appeared a specific WAP signal (Supplementary Fig. S4a).

The antimicrobial activities of WAP protein extract were tested against several Gram-positive (G^+^) and Gram-negative (G^−^) bacteria and fungi by solid-phase assay and minimum concentration (MIC) analysis. The results of the solid-phase assay showed that WAP protein extract (1 mg/mL) had strong antimicrobial effect against tested bacteria with diameters of inhibition zones similar to that with the positive ampicillin control (1 mg/mL), whereas control protein extract (1 mg/mL) and Luria Bertani medium (LB) had no effects on all tested bacteria (Table 3). Surprisingly, the WAP protein extract retained its antibacterial activity after hot or acid treatment (Supplementary Fig. S4b,c). The MICs obtained with WAP protein extract were summarized in Table 4, which were approximately 1–2 mg/mL (equivalent to the protein content with 30–60 μg/mL). The WAP protein extract had broad-spectrum antimicrobial activity against G^+^ and G^−^ bacteria even against *MRSA*, and the WAP protein extract seemed to be more effective than ampicillin.

**Table 3:** The diameters of inhibition zones of WAP protein extract against bacteria (mm).

**Bacteria**	**Ampicillin**	**WAP protein extract**	**Control**	**LB**
*Staphylococcus aureus*	35.0	36.0	0	0
*Micrococcus luteus*	30.0	31.0	0	0
*Bacillus subtilis*	33.0	32.5	0	0
*Bacillus megaterium*	28.0	29.0	0	0
*Escherichia coli*	25.0	24.0	0	0
*Pseudomonas aeruginosa*	32.3	31.5	0	0
*Klebsiella pneumoniae*	33.5	33.5	0	0
*Bacillus thuringiensis*	32.0	31.0	0	0
*MRSA*	20.0	20.0	0	0

**Table 4:** The MIC of WAP protein extract against bacteria.

**Bacteria**	**WAP protein extract (mg/mL)**
*Staphylococcus aureus*	1
*Micrococcus luteus*	2
*Bacillus subtilis*	1
*Bacillus megaterium*	1.5
*Escherichia coli*	2
*Pseudomonas aeruginosa*	2
*Klebsiella pneumoniae*	2
*Bacillus thuringiensis*	2
*Pichia pastoris*	1.5

### Monitoring the protease inhibitory activity of WAP protein extract

**Figure 3:**
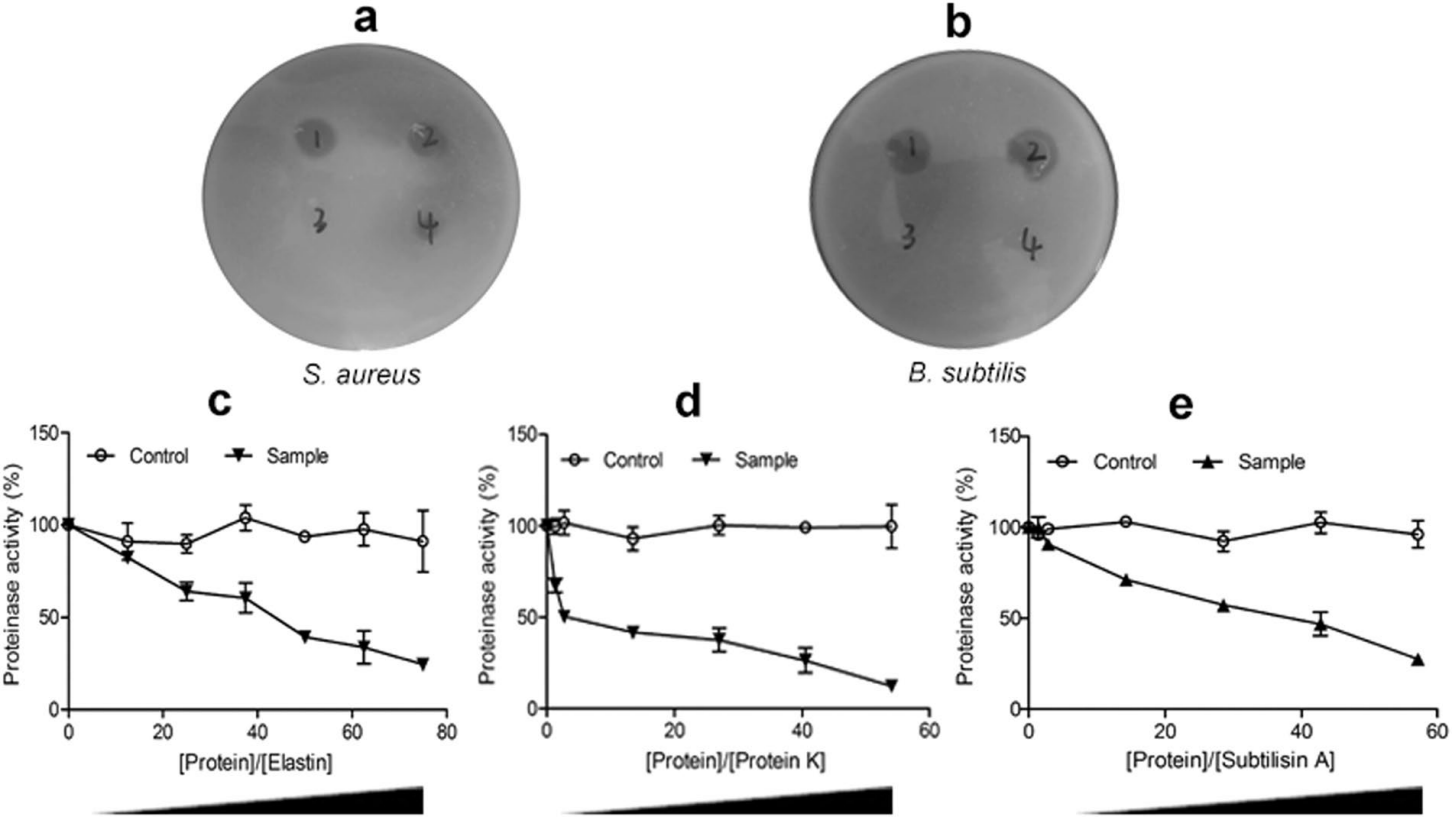
Protease inhibitory activity of WAP protein extract. (**a**,**b**) Inhibitory activities of WAP protein extract against the excretive proteases of *S*. *aureus* and *B*. *subtilis*. The single colony of *S*. *aureus* or *B*. *subtilis* was covered with a paper disc soaking 10 μL different samples. 1: LB, 2: control protein extract (1 mg/mL), and 3–4: WAP protein extracts (4 and 1 mg/mL). (**c–e**) Inhibitory effects of WAP protein extract against commercial proteases of elastase, proteinase K, and subtilisin A. The remaining proteinase activities on the appropriate chromogenic substrate were calculated, and the control protein extract was used as a control.

The protease inhibitory activity of WAP protein extract against proteases derived from *Bacillus subtilis* and *S*. *aureus* was investigated by a disc diffusion technique. After incubation at 28 °C overnight, no transparent zones formed around the paper discs with WAP protein extracts on the plates. By contrast, transparent zones were visible around the paper discs with control protein extract and LB on the plates (Fig. 3a,b), which indicated that WAP protein extract, in contrast to the control protein extract and LB, had a inhibitory effect on proteolytic degradation of proteases derived from *B*. *subtilis* and *S*. *aureus*. To further confirm the potential protease inhibitory activity of WAP protein extract, a few commercial proteases, including elastase, subtilisin A, and proteinase K, were co-incubated with WAP crude extract *in vitro*. The remaining proteinase activities were determined and plotted against the concentrations of the inhibitor. Compared with control protein extract, WAP crude extract exhibited a high level of inhibition on the hydrolysis of N-succinyl-Ala-Ala-Pro-Phe-*p*-nitroanilide in a dose-dependent manner (Fig. 3c–e).

### Assessment of the anti-inflammation effect of WAP protein extract on loach model

**Figure 4:**
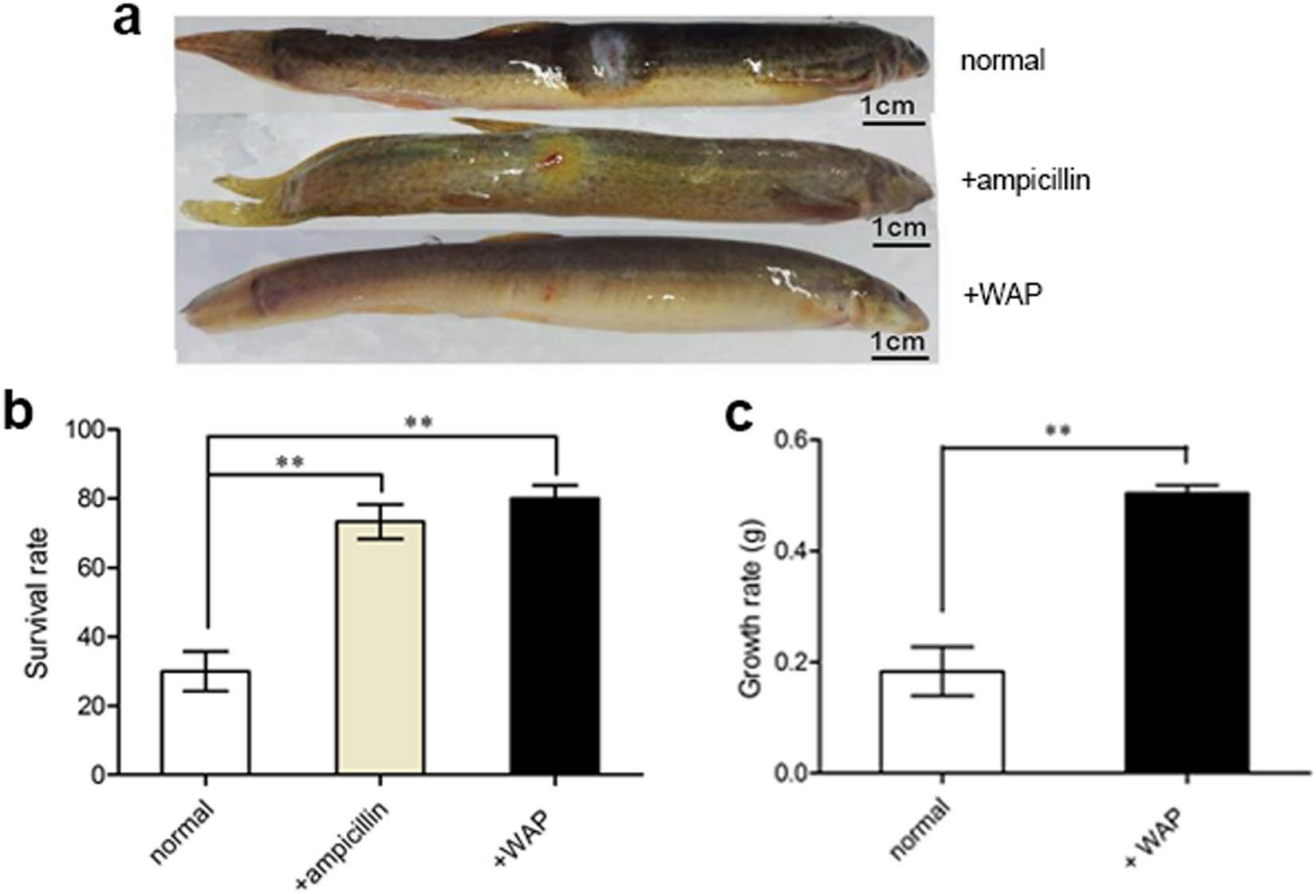
Anti-inflammation effects of WAP protein extract as a feed additive in loach culture. (**a**) WAP protein extract promoted the inflammation recovery of loaches. Exactly 7 days after *S*. *aureus* infection and feeding with different diets, loaches were photographed. Normal: only received normal food, +ampicillin: received normal food plus 3000 mg/kg ampicillin, and +WAP: received normal food plus 3000 mg/kg WAP protein extract. (**b**) WAP protein extract improved the survival rates of loaches. At the 7th day after infection and feeding with different diets, the survival rates of loaches were measured in each treatment group. (**c**) WAP protein extract promoted loaches to put on weight. The growth rate was assayed 20 days after WAP protein extract being fed as feed additive in loach culture. The normal group was used as the control. The asterisks indicate significant differences (**p < 0.01).

*S*. *aureus* is a virulent pathogen most commonly responsible for superficial and invasive skin and soft tissue infections^23^. To test whether WAP protein extract exerted anti-inflammatory functions, two methods (feeding and injection) were used in loach (*Misgurnus anguillicaudatus*) culture. At 1 day post-infection, the *S*. *aureus*-challenged loaches developed typical symptoms of inflammation in the inoculated areas with edema, infiltration, and severe cutaneous erythema. After 7 days of feeding with different foods, inflammation symptoms significantly reduced to hardly observable in normal food plus WAP protein extract (3000 mg/kg) group, as did the normal food plus ampicillin (3000 mg/kg) group with some levels of erythema remaining compared with severe erythema and edema in the normal food group (Fig. 4a). In addition, the normal plus WAP protein extract group maintained the highest survival rate of 80% compared with the normal group with 30% and normal plus ampicillin group with 73.3% (Fig. 4b). Interestingly, the promoting growth activity of WAP protein extract served clearly as a feed additive in loach culture. Results showed that the growth rate of survived loaches in experimental group after 20 days of feeding with normal plus WAP protein extract (3000 mg/kg) was twice more than that of control group fed with normal food (Fig. 4c), and the experimental group had a low mortality rate of 3.3%, only for 1/9 of control group (30%). Taken together, these results confirmed that WAP protein extract, as a feed additive, had strong effects, including anti-inflammation and improved survival rate and growth rate, in loach culture.

**Figure 5:**
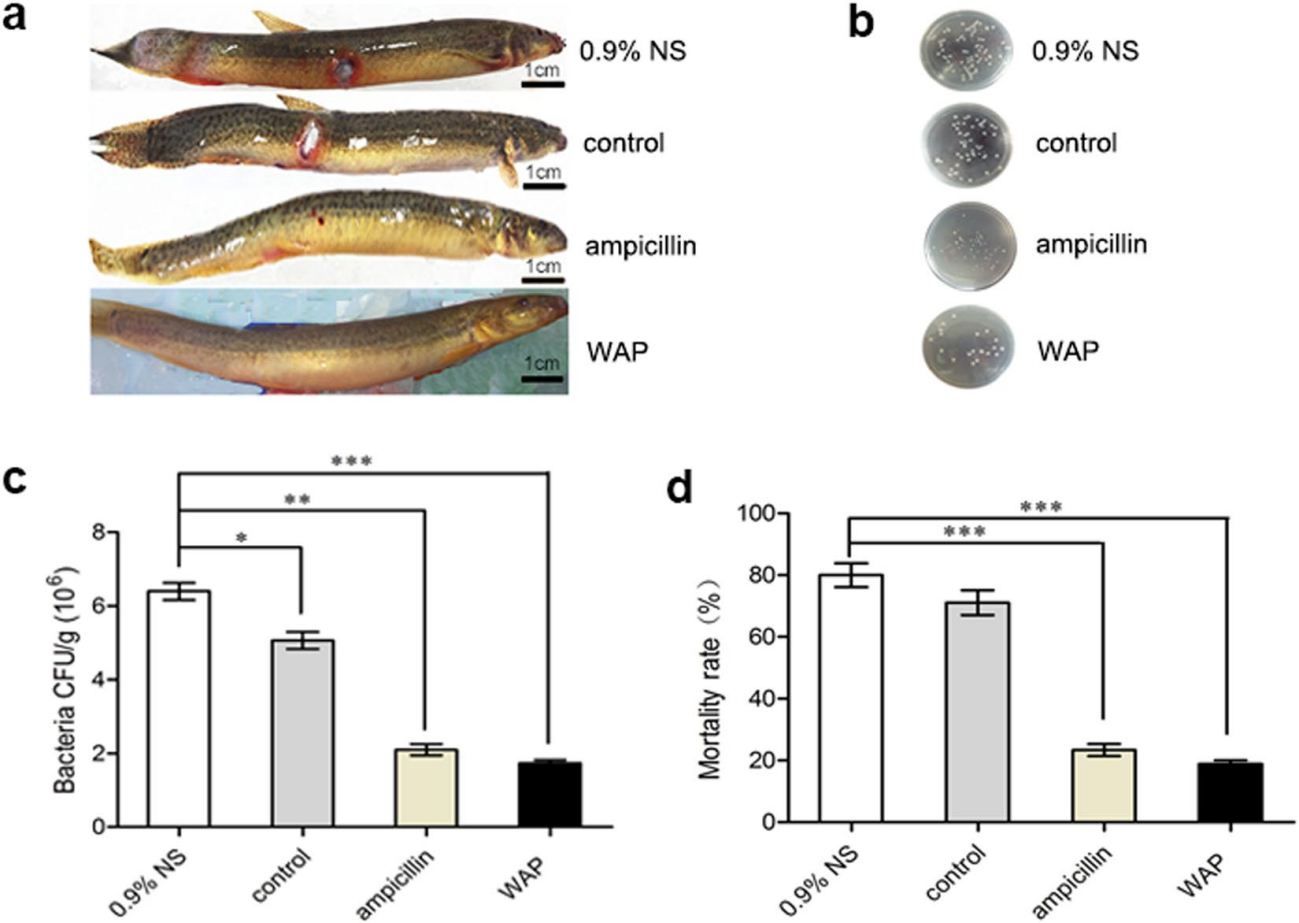
Anti-inflammation effects of WAP protein extract as a drug against bacterial infected loaches. (**a**) On the 5th day, representative images were taken from loaches infected by *S*. *aureus* (1.13 × 10^9^ cfu/loach) and treated with 10 μL of 0.9% NS, control (1 mg/mL control protein extract), ampicillin (1 mg/mL), and WAP (1 mg/mL WAP protein extract). (**b**,**c**) Bacterial colonies and counts of the wound tissues in survived loaches at the 5^th^ day of post infection/treatment. (**d**) The mortality rates of loaches in different groups were calculated within 5 days of post injection/treatment. Data represented mean ± SD from three independent repeats, and the asterisks represent significant differences (*p < 0.05; **p < 0.01; ***p < 0.001, calculated by paired sample *t* test).

To further develop the clinical application of WAP protein extract, loaches as above were subcutaneously inoculated with *S*. *aureus* and developed topical inflammation. Then, the loaches were divided into four groups to accept different topical treatments, including an injection of 0.9% normal saline (NS), control protein extract (1 mg/mL), ampicillin (1 mg/mL), and WAP protein extract (1 mg/mL), 10 μL per loach. At the 5th day, intramuscular administration of WAP protein extract significantly reduced all inflammation symptoms to almost complete recovery, and ampicillin treatment also induced changes in the physical appearance that were very similar to WAP protein extract administration, with an observable red dot. Meanwhile, the treated skin by 0.9% NS and control protein extract exhibited a large extent of erythema and edema (Fig. 5a). On day 5, the average bacterial loads were detected in one inflammatory skin of a survived loach treated by 0.9% NS, control protein extract, ampicillin, or WAP protein extract, which were 6.4 ± 0.26 × 10^6^, 5.1 ± 0.29 × 10^6^, 2.1 ± 0.20 × 10^6^, or 1.7 ± 0.11 × 10^6^ cfu/g, respectively (Fig. 5b,c). Interestingly, the mortality rates of loach were consistent with that of bacterial load, which were 80% in 0.9% NS group, 70% in control group, 21% in ampicillin group, and 19% in WAP group (Fig. 5d). These results indicated that WAP protein extract as a drug could protect the bacteria-infected loach from inflammation by bacterial killing and helping it to heal afterwards.

### Assessment of the therapeutic effect of WAP protein extract on mouse model

**Figure 6:**
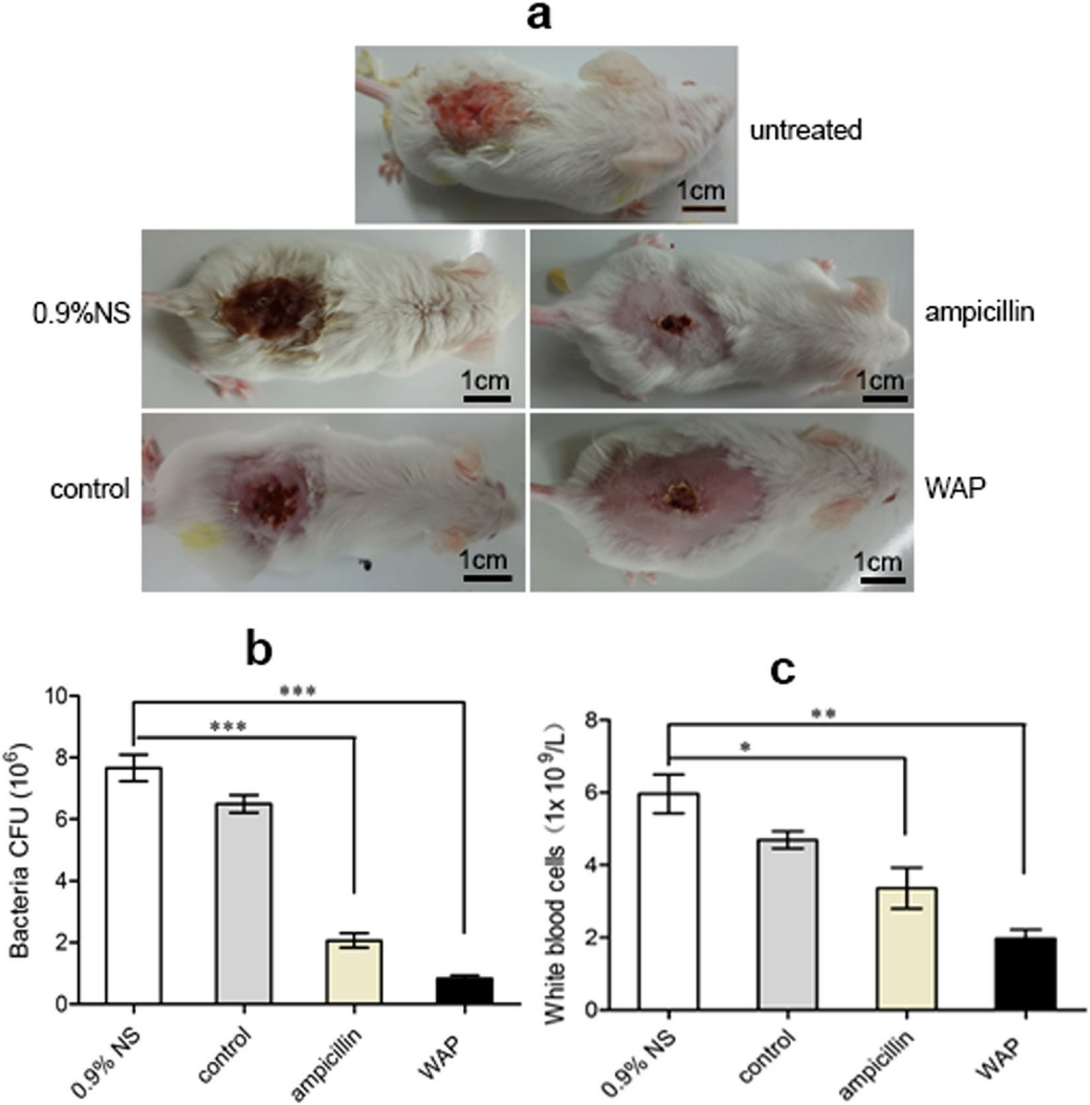
Therapeutic effects of WAP protein extract on mouse model’s wound repair. (**a**) Representative photos of mice. Untreated: the artificial cross incision wound did not receive any treatment at 3 h of post-inoculation with 10 μL of *S*. *aureus* (1 × 10^8^ cfu/mL) (top row). After *S*. *aureus* infection, the wounds received the treatments of 0.9% NS (middle row, right), 3.75 mg/mL control protein extract (bottom row, right), 3.75 mg/mL ampicillin (middle row, left), and 3.75 mg/mL WAP protein extract (bottom row, left) at 100 μL per mouse once a day for 4 days. (**b**,**c**) The bacterial number of the wound tissues and the number of white blood cells in each mouse were determined after 4 days of post treatment. Data were analyzed by the *t* test for paired samples, and the asterisks represent significant differences (*p < 0.05, **p < 0.01, ***p < 0.001).

Four groups of mouse models were inflicted with artificial cross incision wounds (1 cm × 1 cm) and received a sublethal *S*. *aureus* pathogen load, each group with five samples. After being dropped onto the wounded area with four different kinds of agents, including 0.9% NS, ampicillin (3.75 mg/mL), WAP protein extract (3.75 mg/mL), and control protein extract (3.75 mg/mL), daily for 4 successive days at a rate of 100 μL per mouse, the therapeutic effects were assessed by examining the appearance of the lesions. Results showed that the wounds treated by 0.9% NS and control protein extract were not fully closed and became purulent skin erosions, and the color of treated wounds changed into deep, dark red compared with the pink color of untreated wounds. The best therapeutic effect was found in the group treated by WAP protein extract, including the mouse wounds were covered with dry scabs and the edges of the lesion became sharp. Significant improvement was also evident for ampicillin-treated wounds, with a clear base and without any secreted material (Fig. 6a). On the 4th day, after the inflammation assessments, the mice were killed, and scars were removed from the lesions. Wound tissues were homogenized for colony counts. The treatment of WAP protein extract made the wound in a mouse to retain the lowest bacterial load of 0.8 ± 0.11 × 10^6^ cfu/g. The highest bacterial count was from the wound treated by 0.9% NS with 7.7 ± 0.56 × 10^6^ cfu/g, whereas the treatment of control protein extract and ampicillin also reduced wound bacterial number to 6.5 ± 0.33 × 10^6^ and 2.1 ± 0.29 × 10^6^ cfu/g, respectively (Fig. 6b). Compared with 0.9% NS, WAP protein extract had 89% bacteriostatic rate, which was higher than that of ampicillin (73%). In addition, the number of white blood cells of each inflamed mouse was analyzed by a HEMAVET 950 animal blood cell counter on the 4th day. The mouse processed by 0.9% NS had high number of white blood cells, 1.8 and 3 times processed by ampicillin and WAP protein extract, respectively (Fig. 6c). WAP protein extract had strong therapeutic effect on the mouse model’s wound repair.

## Discussion

AMPs are multifunctional effectors of the innate immune system in vertebrates and invertebrates, show antimicrobial activity against a range of pathogenic viruses, bacteria, and fungi, and have been considered potential alternative agents to conventional antibiotics^24^. Despite the high therapeutic potential of AMPs, some drawbacks in terms of poor yield of naturally occurring AMPs and inner toxicity of recombinant AMPs toward *E*. *coli* and yeast have restricted AMPs’ commercial development^6,25^.

WAP domain proteins are important AMPs that exhibit proteinase inhibitory and antimicrobial activities, which have been found in several species of shrimp^21,26–28^. Meanwhile, *F*. *chinensis* WAP as a novel antimicrobial agent has also been verified by transgenic *D*. *melanogaster* in this study.

*D*. *melanogaster* is a model organism with a large set of genetic and molecular tools^29,30^. In addition, the short life cycle, high fecundity, and easy husbandry have made the fruit fly an efficient reactor for the production of massive yields of recombinant proteins in a rapid, cheap, and efficient way^31^. Generation of transgenic flies that overexpress and downregulate gene of interest ubiquitously or in specific tissues is possible by using GAL4/UAS binary expression system in *Drosophila*^32–34^. The pUAST vector had a good effect on the construction of UAS-transgenic *D*. *melanogaster* strains, which not only provided UAS but also provided a mini-white gene that was a marker for insertional mutagenesis; these findings contribute to the feasibility of selecting transgenic *D*. *melanogaster* based on the red-eye phenotype^35^. Therefore, creating hundreds or thousands of transgene insertions in different loci on the genome of transgenic *D*. *melanogaster* with microinjection of recombinant pUAST vector being mediated by PΔ2–3 plasmid was feasible^36^. In the present paper, the *wap*/pUAST construction was made by inserting *F*. *chinensis wap* gene downstream of the UAS sequence. In addition, the microinjection of 500 ng/μL mixed plasmids of *wap*/pUAST and PΔ2–3 achieved about 2% UAS-*wap* transgenic frequency (Table 1).

The GAL4/UAS system has been used to study the spatio-temporal expression and functions of several endogenous and exogenous genes in *D*. *melanogaster* in which target gene was linked to UAS sequences, and Gal4 drove the expression of UAS-transgene^37–39^. We also observed a very high expression level of *wap* gene in the progeny of t6 UAS-*wap* transgenic strain crossing with *actin*-Gal4; the level was higher by 160 times compared with the control (Fig. 1a).

AMPs efficiently combat microbial pathogens^40^. The antibacterial functions of *F*. *chinensis wap* gene in transgenic *D*. *melanogaster* were verified by reducing the death rates of UAS*-wap/*Gal4 flies in standard medium mixed with *E*. *coli*, *S*. *aureus*, and *M*. *luteus*, by increasing the survival rate of UAS*-wap/*Gal4 flies challenged by *E*. *coli*, and by decreasing the microbial load of UAS*-wap/*Gal4 flies compared with UAS*-wap/w*^1118^ and Gal4*/w*^1118^ flies (Fig. 2). Moreover, WAP protein extract made by UAS-*wap*/Gal4 flies showed antibacterial and protease inhibitory activities *in vitro* (Tables 3 and 4 and Fig. 3), which were similar to previous report^21^. The exciting thing is that a special WAP protein band was detected in the WAP protein extract by western blot (Supplementary Fig. S4a). It is reasonable for us to speculate that the activities of WAP protein extract should be mediated by the WAP protein. It should be noted that, maybe due to glycosylation, the molecular weight of the WAP recombinant protein in transgenic *D*. *melanogaster* was larger than that of counterpart in *F*. *chinensis*. Importantly, the effects of weight increase and anti-inflammation of WAP protein extract were proved on loach and mouse models (Figs 4–6).

In this study, the UAS*-wap* transgenic lines were constructed, and high expression of *wap* gene was verified in UAS*-wap/*Gal4 flies. In addition, the overexpression of *wap* gene enabled UAS*-wap/*Gal4 flies with enhanced antimicrobial activity by reducing mortality rates and *in vivo* bacterial load. The dual functions of antimicrobial and antiprotease activities of WAP protein extract prepared with UAS*-wap/*Gal4 flies were confirmed *in vitro*. The therapeutic effects of WAP protein extract were verified based on the responses of the promotion of inflammatory recovery and wound-healing on loach and mouse models. Furthermore, the effects of WAP protein extract as a feed additive for the promotion of loach growth and reducing bacteria load were determined. In conclusion, the *wap* gene had many attractive features as a novel antibiotic reagent, and the UAS*-wap/*Gal4 transgenic *D*. *melanogaster* offered exciting opportunities as a bioreactor of WAP recombinant protein. Results provide a good foundation for the development of WAP to a novel therapeutic medicine in the future.

## Materials and Methods

### *D*. *melanogaster* strains

The *D*. *melanogaster* strains of *w*^1118^, *ppl*-Gal4, *he*-Gal4 were from the laboratory (Stockholm University, Sweden) of Prof. Ulrich Theopold. Additional strains *actin*-Gal4, balancers of 3703 (w−/w−;Sco/Cyo;Sb/Tb), and FM7a (Bar/Bar;+/+;+/+) were kindly gifted by Prof. Li Liu (Institute of Biophysics, Chinese Academy of Sciences, China). All flies were raised on cornmeal agar media at 18 °C for preservation and 25 °C for breeding, 55 ± 5% humidity, and 12 h light/12 h dark cycle.

### Construction of UAS-*wap* transgenic *D*. *melanogaster*

The cDNA segment (ORF, 273 bp) of *F*. *chinensis wap* gene was amplified by PCR with primers of *wap* F and *wap* R from *wap*/pGEM-T Easy plasmid from Prof. Jinxing Wang of the Shandong University in China using *Pfu* DNA polymerase (Fermentas). The PCR reaction included predenaturation at 94 °C for 2 min, 33 cycles of 94 °C for 30 s, 61 °C for 45 s, 72 °C for 1 min, and an extension at 72 °C for 10 min. PCR products were separated by gel electrophoresis, and the *wap* fragment was purified by DNA purification kit (Qiagen, Germany) and subjected to directional subcloning into pUAST vector at Kpn I and Xba I sites to form the recombinant plasmid of *wap*/pUAST.

The *wap*/pUAST plasmid was mixted with PΔ2–3 helper plasmid according to 3:1 ratio and then extracted with phenol-chloroform. On the day of microinjection, *w*^1118^ eggs in a 30 min egg-laying period were picked up, and the DNA mixture of *wap*/pUAST together with PΔ2–3 plasmids was microinjected into the germ cells of *w*^1118^ embryos according to a standard protocol^36^. The injected embryos were placed in a small Petri dish in a humid chamber until being hatched to larvae at 18 °C on the third day.

The newly hatched larvae were transferred to a vial with fresh food and grown to G0 adults on the 12th day at 25 °C. G0 males and G0 virgins were collected to cross with *w*^1118^ flies by 2♂:2☿ ratio. Each G1 offspring with pigmented eyes in single crossing vial was selected as the UAS-*wap* transgenic strain. The eye color of these transgenic flies varied from light red to dark red depending on the insertion site of the *wap* gene. For simplicity, we generally referred to the transgenic flies as “red-eyed”. To verify the UAS-*wap* transgenic strains carrying *wap* gene, their genomic DNAs together with those of controls including *w*^1118^, *ppl*-Gal4, *he*-Gal4, and *actin*-Gal4 were extracted using DNA extraction kit (Qiagen, Germany). PCR was carried out using the gDNA templates and *wap* F and *wap* R primers. PCR products were separated with agarose gel electrophoresis.

### Mapping of *wap* gene

Based on the phenotype analysis of offspring, the *wap* gene was mapped to a given chromosome in UAS-*wap* transgenic line. According to the mapping procedure (Supplementary Fig. S3), one red-eyed male (♂UAS-*wap*) was selected from a transgenic line to cross with one white-eyed 3703 balancer virgin females (☿w−/w−;Sco/Cyo;Sb/Tb). In F1 generation, if all males were white-eyed and all females were red-eyed, the *wap* gene was located at chromosome X in the transgenic line; if some of males and females were red-eyed and the others were white-eyed, the *wap* gene was either located at chromosome II or chromosome III in the transgenic line. Then, the males with distinct phenotypes, including warped-wing and shorten-stubble (+/Cyo;+/Tb;red-eyed) or warped-wing and hairy-shoulder (+/Cyo;Sb/+; red-eyed), were selected from the F1 generation of the transgenic line to set the second round of crosses with 3703 balancer virgin females individually. If the phenotypes of warped-wing, hairy-shoulder, and shorten-stubble (+/Cyo;Sb/Tb;red) appeared in the F2 generations, the *wap* gene was inserted into chromosome II in the transgenic line. Similarly, when the phenotypes of hirsutulous-back, warped-wing, and hairy-shoulder (Sco/Cyo;+/Sb;red-eyed) appeared in the F2 generation, the *wap* gene was inserted into chromosome III in the transgenic line. Thus, when multiple phenotypes, including warped-wing and hirsutulous-back (Sco/Cyo;red-eyed) or hairy-shoulder and shorten-stubble (Sb/Tb;red-eyed), appeared in the F2 generation, the *wap* gene should be doubly inserted into chromosome II and III in the transgenic line.

### Expression analysis of *wap* gene in transgenic *D*. *melanogaster*

The UAS-*wap* lines crossed with *actin*-Gal4 line to generate the hybrid offspring of UAS-*wap/*Gal4 that constituted experimental group. Meanwhile, the UAS-*wap* line and *actin*-Gal4 line individually crossed with *w*^1118^ line to generate their hybrid offspring of UAS-*wap*/*w*^1118^ and *w*^1118^/Gal4 that constituted two different control groups, respectively. The newly-emerged adults for 3 days in experimental and control groups were individually collected and divided into two parts at 1:1 ratio of males and females. The first part remained normal, and the second part accepted an infection by pricking its post-abdomen with a sterile insect needle previously dipped into a bacterial suspension of *E*. *coli* (1.5 × 10^9^ cfu/mL). After 24 h, total RNAs were isolated using the normal and bacteria-challenged samples with TRIzol reagent (Invitrogen, USA). cDNAs were synthesized by the instructions of the SMART cDNA Synthesis kit (BD Bioscience Clontech, USA) using the total RNAs and primers of Oligoanchor R and Smart F. qRT-PCR was carried out to evaluate the gene expression changes of *wap* and known *D*. *melanogaster* AMP genes, including *attA*, *attB*, *dpt*, *def*, *dro*, *ce-A1*, and *ce-A2* in experimental and control samples with a CFX96 Real-Time System (Bio-Rad, USA) and iQSYBR Green Supermix (Bio-Rad, USA) following previously described methods^41^. The quantitative and qualitative control was *β-actin* or *rp49* that was used to normalize the variation in the amount of cDNA in each reaction. The qRT-PCR included 1 cycle at 95 °C for 10 min; 40 cycles at 95 °C for 15 s, 60 °C for 50 s, and 72 °C for 2 s; and a melting period from 65 °C to 95 °C. The 2^−ΔΔCT^ represents the relative expression level of target gene in which the Ct discrepancy of control to target gene was calculated as ΔΔCt = Ct_*control*_ − Ct_*gene*_. All samples were analyzed in triplicate during the qRT-PCR. Data were analyzed by the unpaired *t*-test, and *p* < 0.05 indicated statistical significance.

### Assessment of antibacterial activity of *wap*-expressing transgenic *D*. *melanogaster*

The parent crosses, including the UAS-*wap* transgenic lines crossing with *actin*-Gal4 line to generate the progeny of UAS-*wap/*Gal4 in experimental group, and the UAS-*wap* lines and *actin*-Gal4 line individually crossing with *w*^1118^ to generate the progeny of UAS-*wap*/*w*^1118^ and *w*^1118^/Gal4 in control groups, were raised on standard cornmeal/agar medium in the presence of *E*. *coli*, *S*. *aureus*, and *M*. *luteus* at 25 °C until their F1 generation reached adulthood. The *E*. *coli*, *S*. *aureus*, and *M*. *luteus* (obtained from the School of Life Sciences, Shandong University, China) were cultured overnight in LB medium (1% tryptone, 0.5% yeast extract, 1% NaCl), collected by centrifugation at 5,000 × g for 5 min, washed by sterile PBS (137 mM NaCl, 2.7 mM KCl, 10 mM Na_2_HPO_4_, 2 mM KH_2_PO_4_, pH7.4) thrice, re-suspended in PBS, plated for colony counting (using a hemocytometer and following serial dilution), mixed according to 1:1:1 ratio, and added into culture medium to 1.2 × 10^7^ cfu/mL. Counting newly emerged F1 flies every day, the cumulative mortalities were evaluated on F1 flies in each group within 12 days of post-eclosion. Additionally, the anaesthetized experimental and control male flies were challenged by pricking the flies’ postabdomen with a sterile insect needle previously dipped into a bacterial suspension of *E*. *coli* or *S*. *aureus* or *M*. *luteus* (1.5 × 10^9^ cfu/mL). Meanwhile, the PBS treatment of experimental flies as negative control was mocked. The number of dead flies was recorded daily for each group, and survival rates during 6 days of post-injury were calculated. For bacterial counts, the survivors in experimental and control groups were individually collected and ground in PBS. After being diluted to appropriate concentration, the homogenates of 100 μL in each group were plated onto LB agar plates (1% tryptone, 0.5% yeast extract, 1% NaCl, and 1.5% agar). The plates were placed at 37 °C for 24 h, and the bacterial colonies were counted for each plate. All experiments were repeated thrice. The results were expressed as the mean ± SD (standard deviation) for 3 independent repeats using Graph-Pad Prism^42^. The statistical significance (***p < 0.001, **p < 0.01, *p < 0.05) was analyzed by *t*-test.

### Monitoring the antimicrobial activity of WAP protein extract

For getting WAP protein extract and control protein extract, the UAS-*wap*/Gal4 experimental flies and control flies of UAS-*wap/w*^1118^ and *w*^1118^*/*Gal4 were separately soaked for 4–5 h by ammonium acetate buffer (0.05 mol/L, pH 5.5) according to 1:5 ratio (g/mL) and then homogenized under ice bath. The homogenate was boiled for 10 min in water bath, and its supernatant was collected by centrifugation at 12,000 × g for 20 min at 4 °C. After lyophilization, protein extracts were stored at −20 °C. When being used, these extracts were adjusted to appropriate concentration with sterile water, filtered through 0.22 μm filter, and quantified by the Bradford method^43^. In addition, the thermal stability and acid resistance of WAP protein extract were detected by solid-phase assay using boiled samples for 5, 10, 15, 20, and 30 min and acid-treated samples by hydrochloric acid of pH 1, 2, 3, and 4 for 30 min.

For detecting the WAP protein in WAP protein extract, the western blot analysis was employed using WAP antiserum against WAP protein extract and control protein extract. The WAP antiserum was prepared from rabbit via traditional method using purified WAP recombinant protein. The expression and purification of WAP recombinant protein were carried out as previously described^21^. Briefly, the cDNA fragment of WAP whole peptide was amplified using primers of *wap-EX* F and *wap-EX* R (Table 2), and then ligated with pGEX-4T-1 vector by EcoR I and Xho I sites to form the *wap*/pGEX-4T-1 plasmid. The recombinant WAP protein was expressed in the form of inclusion bodies in *wap*/pGEX-4T-1/*E*. *coli*. After being denatured and renatured, the WAP recombinant protein was purified by Glutathione Sepharose 4B affinity gel column as per manufacturer’s instructions (Pharmacia).

The antimicrobial activity of WAP protein extract was investigated against G^+^ bacteria (*Bacillus megaterium*, *B*. *subtilis*, *Bacillus*. *thuringiensis*, *S*. *aureus*, and *M*. *luteus* and G^–^ bacteria (*Klebsiella pneumonia*, *E*. *coli*, and *Pseudomonas aeruginosa*), as well as *MRSA in vitro* using solid-phase assay and MIC measurement method as described previously^44–47^. The solid-phase assay was performed as follows. The bacterial cultures were grown up to OD_600_ of 0.5 in LB medium, centrifuged at 6000 g for 5 min to collect the bacteria pellets, and adjusted to about 10^5^ cfu/mL concentrations. The bacteria were mixed with warm PB (LB free of yeast extract) and poured onto a Petri dish to prepare agar plates. Each plate was covered equidistantly with 4 pieces of 4.0 mm in diameter of filter papers that were saturated in advance with 10 μL filtered WAP protein extract (1 mg/mL), control extract (1 mg/mL), ampicillin (1 mg/mL), and LB. After overnight culture at 37 °C, the pieces of filter paper were carefully removed, and the antimicrobial activities were determined by measuring the inhibition zones on PB plates. All tests were performed in duplicates, and inhibition zones were recorded in mm. To determine the MIC of WAP protein extract, the cultured mid-logarithmic phase bacteria were collected and diluted with sterile PBS to 10^5^ cfu/mL concentration. The WAP extract at 2-fold dilutions (50 μL) was added to the 96-well plate containing 100 μL bacteria per well, and the plate was cultured for 24 h at 28 °C. Bacterial growth was measured at an absorbance of 600 nm using ELX800 Universal Microplate Reader (Bio-Tek, USA).

### Detecting the protease inhibitory activity of WAP protein extract

The protease inhibitory activity of WAP protein extract against secreted bacterial proteases and commercial proteases was detected following previously described methods^26,48,49^. Briefly, a single colony of *B*. *subtilis* or *S*. *aureus* was transferred from an LB plate to a skim milk plate (skim milk plus agar, 1% each) with a sterilized toothpick and covered with a piece of 4.0 mm filter paper disc. Then, 10 μL of different sterile-filtered samples, including LB, control protein extract (1 mg/mL), and WAP protein extracts (4 and 1 mg/mL), was added onto the paper disc. After overnight culture at 28 °C, the pieces of filter paper were carefully removed, and the transparent zones were measured on the skim milk plates. In addition, three commercial proteases, including elastase, subtilisin A, and proteinase K (Sigma, St. Louis, MO, USA), and their substrate N-Succinyl-Ala-Ala-Pro-Phe-*p*-nitroanilide (Sigma, St. Louis, MO, USA) was used to determine the inhibitory activity of WAP protein extract. The reaction mixture (100 μL) contained 100 mM Tris-HCl, pH 8.0; 1 µM N-Succinyl-Ala-Ala-Pro-Phe-*p*-nitroanilide; and 80 nM elastase/3.7 nM proteinase K/3.5 nM subtilisin A. The assay of the residual enzyme activity was followed by the addition of WAP protein extract of 1, 2, 3, 4, 5, and 6 µM for elastase and 0, 0.005, 0.010, 0.050, 0.100, 0.150, and 0.2 µM for proteinase K and subtilisin A. The resulting mixture was allowed to settle for 15 min at 37 °C and then terminated by the addition of 50 µL 50% acetic acid. The absorbance was measured at 405 nm using ELX800 Universal Microplate Reader (Bio-Tek, USA). The same mass of control extract was substituted for WAP protein extract as a negative control. The experiments were performed in triplicate for statistical analysis.

### Assessment of the anti-inflammation effect of WAP protein extract on loach model

*M*. *anguillicaudatus* were purchased from a market in Jinan city in the Shandong province of China, and maintained in an oxygenated, recirculating and ambient freshwater system. All experimental protocols followed the People’s Republic of China Laboratory Animal Management Regulations issued by the State Council of China and were implemented in University of Jinan under the permission of the Committee of Shandong Experimental Animal Management. Two methods of feeding and injection were used for the anti-inflammation activity assay of WAP protein extract. For the feeding, healthy loaches subcutaneously inoculated *S*. *aureus* (1.13 × 10^9^ cfu/loach) with a 1 mL sterile syringe and then divided into three groups, with 30 loaches in each group, 21 to 23 g per loach. The first group was continuously fed with normal diet, normal plus ampicillin diet (3000 mg/kg) to the second group, and normal plus WAP protein extract (3000 mg/kg) diet to the third group. The inflammatory status and the number of dead loaches in each group were monitored daily for 7 days of post-inoculation. For the injection method, healthy loaches were divided into four groups (30 loaches per group, 21–23 g per loach) and received an inoculation of *S*. *aureus* as above at 6 h post-infection, followed by the injection of 0.9% NS, control extract (1 mg/mL), ampicillin (1 mg/mL), and WAP protein extract (1 mg/mL) at 10 μL per loach. In 5 days post-injection, the inflammatory symptoms and the dead loaches in each group were monitored and photographed. Meanwhile, the inflammatory tissues (0.1 g) were collected and washed twice with 0.9% NS, homogenized in 1 mL 0.9% NS, and diluted 1000 times. Exactly 100 µL of homogenates was plated onto an LB plate (1% tryptone, 0.5% yeast extract, 1% NaCl, and 1.5% agar) and cultured at 37 °C for 18 h, and the colony counts were recorded. All experiments were repeated at least thrice, and the data were statistically analyzed using the Student *t*-test.

The promoting growth activity of WAP protein extract was tested in loach cultures. First, healthy loaches were randomly divided into experimental and control groups (120 loaches per group, 11 ± 1 g per loach). Each group was divided into three subgroups, 40 loaches per subgroup. The experimental group was fed with normal diet supplemented with WAP protein extract (3000 mg/kg), whereas the control group was fed with normal diet. After 20 days, the surviving samples were weighed.

### Assessment of the therapeutic effect of WAP protein extract on mouse model

Kunming mice (8 weeks old, 23–25 g weight) purchased from the Experimental Animal Center of Shandong University in China were used to investigate the therapeutic effect of WAP protein extract. All experimental protocols followed the People’s Republic of China Laboratory Animal Management Regulations issued by the State Council of China and were implemented in Shandong Monitoring Center for Experimental Animals under the permission of the Committee of Shandong Experimental Animal Management. Mice were housed in an independent air isolation cage (30 × 20 × 15 cm, one in each cage) with food and water ad libitum. They were allowed to habituate the experimental environment of 12 h light/dark cycles at 23 ± 1 °C for a week to reduce any stimulation that arose from the new environment. At 1 day before surgery, hair removal was performed in the back area with 5% sodium sulfide. Subsequently, the inflammatory model was established by dropping 10 μL of *S*. *aureus* (1 × 10^8^ cfu/mL) to a cross incision (1 cm × 1 cm) cut by a scalpel in the mouse back skin after anesthetizing with an intraperitoneal injection of amobarbital (1%, 45 mg/kg body weight) (Vedco, Inc., St. Joseph, MO, USA). At 3 h post-inoculation, the mice were divided into four groups randomly. Each group had five samples. Afterward, we dropped 0.9% NS, ampicillin (3.75 mg/mL), WAP protein extract (3.75 mg/mL), and control extract (3.75 mg/mL) onto the wounded areas at 100 μL per mouse. The mice were observed daily and photographed for 4 successive days. On the fourth day, after the inflammation assessment, the mice were immediately anesthetized, and 1 mL blood was extracted from mouse heart using a sterile syringe with additional 1 mL anticoagulant buffer (EDTA-K_2_, 1 mg/mL). The number of white blood cells of each sample was analyzed by a HEMAVET 950 animal blood cell counter. Subsequently, mice were killed by cervical dislocation, and the inflammatory muscular tissues (0.1 g) of the wounds were isolated for bacterial counting as above.

## Electronic supplementary material

Supplementary Information
