# Supplementary material for: Expression of the Shrimp wap gene in Drosophila elicits defense responses and protease inhibitory activity
Source: Sci Rep. 2018 Jun 8;8:8779. doi: 10.1038/s41598-018-26466-6 (PMC5993750; doi:10.1038/s41598-018-26466-6)
Supplement: Supplementary file 1 — Supplementary Information [file 41598_2018_26466_MOESM1_ESM.doc]

**Supplementary Information**

Expression of the Shrimp*wap* gene in *Drosophila* elicits defense responses and protease inhibitory activity^＊^

Dianxiang Li^1,^ *, Yuanyuan Luan^1,+^, Lei Wang^1,+^, Mei Qi^1,+^, Jinxing Wang^2^, Jidong Xu^2^, Badrul Arefin^3^, and Meixia Li^4^


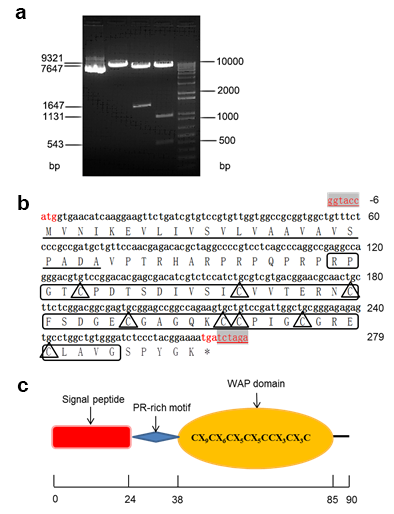


**Supplementary Fig. S1.** Construction of *wap*/pUST recombinant vector. (**a**) The digestion results of *wap*/pUAST with BamH І and Kpn І. The pUAST vector (9050 bp in length) contained two BamH I restriction sites (3013, 4416), one Kpn I site (3556) and one Xba I site (3558). The *wap*/pUAST construct (Lane 1) became a single fragment of 9321 bp post-digestion with Kpn I (Lane 2), and digested with BamH I to produce two fragments (7647 bp and 1674 bp, Lane 3), and double digestion with Kpn I and BamH I to produce two new fragments of 1131 bp and 543 bp which resulted from the 1674 bp fragment digested by Kpn I (Lane 4). Lane 5: 10 kb marker. (**b**) The sequencing results of *wap*/pUAST showed the nucleotide and deduced amino acid sequence of the open reading frame of *F. chinensis* *wap* gene. The predicted signal peptide is underlined. A putative WAP domain is shown in black box. The N-terminal of the *wap* gene contains a Kpn І site (ggtacc, gray shadow). The asterisk (*) indicates the stop codon (tga) followed by an Xba I site (tctaga, gray shadow). The identical cysteines that characterize WAP domain are in the triangles. The nucleotides are numbered in the right. (**c**) Scheme of the WAP structure. The N-terminal, middle and C-terminal of the WAP were denoted as signal peptide, proline enrichment region (PR-rich motif) and WAP domain, respectively.

**
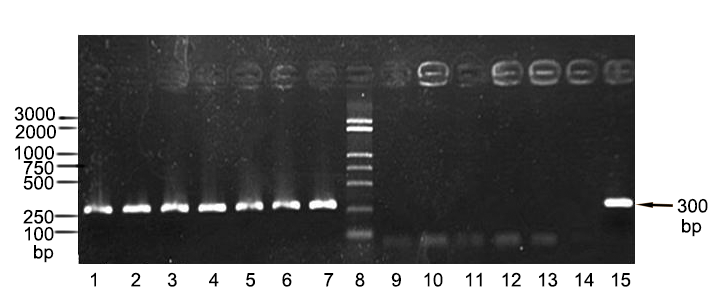
**

**Supplementary Fig. S2.** The UAS-*wap* transgenic *D. melanogaster* identified by PCR with *wap*-specific primers (*wap* F and *wap* R). Lane 1-7, the insertion of *wap* gene was confirmed by PCR using gDNAs of t6, t78, tx5, tx5, t10, t10, and t32 UAS-*wap* transgenic lines, respectively; Lane 8, DL3000 marker; Lane 9-13, the *wap* gene had not be detected by PCR from the gDNAs of *w^1118^*, *actin*-Gal4, *he*-Gal4, *ppl*-Gal4, *elav*-Gal4, respectively; Lane 14, PCR negative control; Lane 15, PCR positive control using *wap*/pUAST vector as the template, the 300 bp *wap* band shown by arrow.


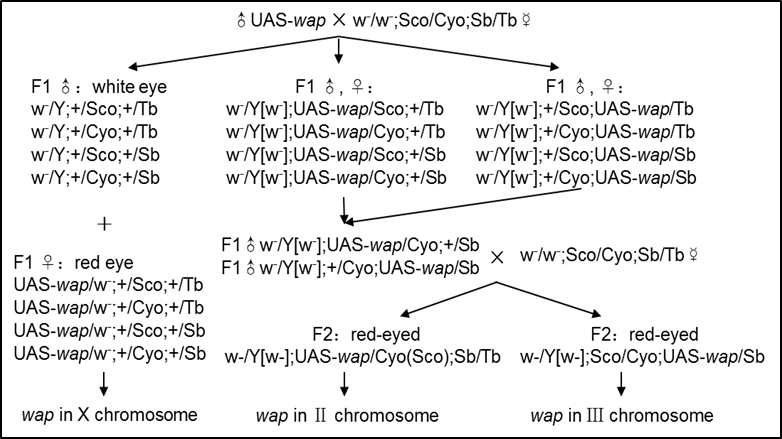


**Supplementary Fig. S3.** The chart of *wap* gene mapping


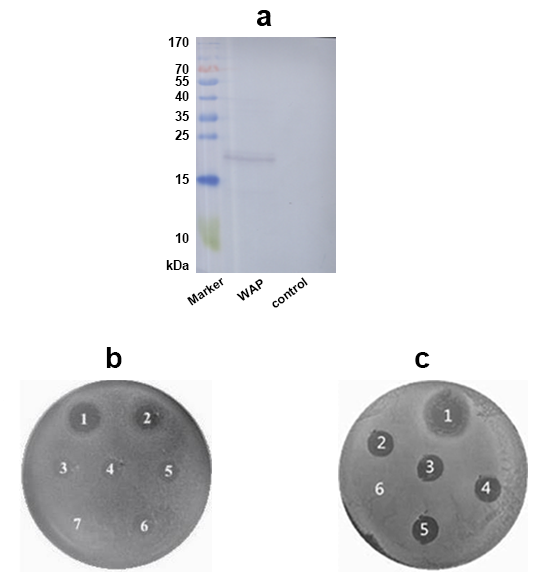


**Supplementary Fig. S4.** The antibacterial properties of WAP protein extract. (**a**) This was the results of western blot analysis with WAP antiserum from rabbits against WAP protein extract (WAP) and control protein extract (control). (**b**) The antibacterial activity of hot treated-WAP protein extract against *S. aureus*. The sign of 1, 2, 3, 4, 5, and 6 stood for the normal and boiled WAP protein extract (each with 1 mg/mL) by boiling bath for 5, 10, 15, 20, and 30 min, respectively. The sign of 7 stood for sterile water as negative control. (**c**) The antibacterial activity of acid treated-WAP protein extract against *S. aureus*. The sign of 1, 2, 3, 4, and 5 stood for the normal and processed WAP protein extract (1 mg/mL) by hydrochloric acid of pH 1, 2, 3, and 4 for 30 min, respectively. The sign of 6 stood for sterile water as negative control.
